# Age-associated B cells and double-negative B cells: two sides of the same coin? The answer depends on the context

**DOI:** 10.3389/fragi.2026.1752452

**Published:** 2026-01-21

**Authors:** Malamatenia Lamprinou, Athanasios Sachinidis, Theodoros Dimitroulas

**Affiliations:** 1 School of Medicine, Aristotle University of Thessaloniki, Thessaloniki, Greece; 2 4th Department of Internal Medicine, Hippokration General Hospital of Thessaloniki, Thessaloniki, Greece

**Keywords:** age-associated B cells, aging, atypical B cells, autoimmunity, chronic infections, chronic inflammation, double-negative B cells, immunosenescence

## Introduction

B cells are central players in adaptive immunity, orchestrating humoral responses through antibody production, antigen presentation, and cytokine secretion. During aging, cumulative immunological changes reshape the B-cell compartment in a process termed immunosenescence, which has profound consequences for infection control, vaccine efficacy, and susceptibility to autoimmunity (Frasca and Blomberg, 2011; Cancro, 2020). Among the most striking features of B-cell remodeling is the expansion of atypical B cell subsets, such as age-associated B cells (ABCs) and double-negative (DN) B cells. Both subsets have become focal points of intense research, because of their enrichment in elderly individuals, their expansion in autoimmune diseases, and their presence in chronic infections (Riley et al., 2017; Sachinidis et al., 2020; Hao et al., 2011; Sachinidis and Garyfallos, 2021). Yet, their precise lineage relationships remain controversial. Some evidence suggests that DN2 cells, which refer to an extrafollicular DN sub-population (Jenks et al., 2018), may overlap functionally and transcriptionally with ABCs, raising the central question: are ABCs and DN B cells two sides of the same coin, or distinct entities? Clarifying their identity is complicated further by nomenclature inconsistencies across species, tissues, and disease contexts (Jenks et al., 2018; Colonna-Romano et al., 2009; Wang et al., 2018). This manuscript synthesizes current knowledge on ABCs and DN subsets, examining their biology in aging, autoimmunity, and infection, with a focus on their transcriptomic signatures and therapeutic relevance. Taking into account these data, we provide an opinion on whether ABCs and DN B cells should be considered identical populations or two distinct B cell subsets.

## Age-associated B cells (ABCs)

ABCs were first identified in aged mice, as CD19^+^CD21^−^CD23^−^ B cells expressing the transcription factor T-bet and the integrin CD11c (Hao et al., 2011). Their percentages in young healthy individuals are low but steadily increase with age (Hao et al., 2011). In healthy aging, a subset of ABCs exhibits reduced BCR-mediated antibody production, consistent with aspects of immunosenescence, though these cells retain other immune functions (Cancro, 2020). However, their exact role is not yet completely understood.

ABC populations were also reported to expand in autoimmune diseases, as well as in chronic-infections (Wang et al., 2018; Portugal et al., 2017). In the context of autoimmunity, T-bet expression in B cells is elevated, leading to increased antibody production, enhanced antigen presentation to T cells and also formation of germinal centers (GCs), all drivers of immunological reaction (Rubtsov et al., 2017). In the context of infectious diseases, on the other hand, ABCs’ role is less clear. It seems that these cells contribute to pathogen clearance, thus display a protective role (Rubtsova et al., 2015).

Up to this day, the origin of ABCs remains a mystery. It has been shown, though, that these cells express a diverse Ig repertoire, portrayed by somatic hypermutations and antigen-driven activation (Cancro, 2020). Based on these features, a GC–experienced origin for at least a subset of ABCs has been proposed, although alternative hypotheses cannot be excluded. For instance, formation through homeostatic proliferation is also a potential route leading to the generation of ABCs (Cancro, 2020). Moreover, it’s worth mentioning that B cell activation involves, not only GC reactions, but also extrafollicular and other GC-independent pathways (Sachinidis and Garyfallos, 2021).

ABCs exhibit heightened responsiveness to endosomal Toll-like receptor (TLR) signals, particularly TLR7 and TLR9, and can be driven to differentiate by TLR stimulation in combination with IFN-γ and/or IL-21, depending on the experimental context (Cancro, 2020; Wang et al., 2018; Rubtsov et al., 2011; Liu et al., 2024; Naradikian et al., 2016). IL-21 robustly induces CD11c expression, contributing to the ABC phenotype, whereas IFN-γ primarily promotes T-bet expression, reflecting context-dependent effects reported in primary studies (Cancro, 2020; Liu et al., 2024; Naradikian et al., 2016). BCR signaling can also contribute to ABC activation, in conjunction with TLR, IFN-γ, IL-21, and/or CD40 signals, although it is insufficient to drive differentiation on its own (Rubtsov et al., 2017; Imabayashi et al., 2025). Of note, TLR7 is an X linked transmembrane receptor, closely related to ABC activation, clarifying the higher rate of autoimmunity onset in female patients (Sachinidis et al., 2020).

As far as transcriptomic profiling of ABCs is concerned, data derived from mice has shown that ABCs are a unique sub-population of B cells, discrete from B1 and FO B cells. In detail, ABCs highly express CD11c and T-bet, along with transcripts of immunoglobulin heavy chain and CD138 (Rubtsov et al., 2011). Intermediate expression of transcription factors involved in plasma cell differentiation has also been reported, thus indicating that ABCs are probably plasma cell precursors (Rubtsov et al., 2011). In the context of autoimmunity, transcriptomic analyses of ABCs have revealed that IL-21 inducible genes, as well as genes associated with cell adhesion, are strongly upregulated (Wang et al., 2018).

In systemic autoimmune diseases, such as systemic lupus erythematosus (SLE), rheumatoid arthritis (RA), Sjögren’s syndrome (SS) and systemic sclerosis (SSc), ABCs expand prematurely and–in some cases - correlate with disease activity, autoantibody titers, and organ involvement (Sachinidis et al., 2020; Bagavant et al., 2024; Kourkouni et al., 2024). More specifically, T-bet + B cells (which are considered as ABCs) are expanded in SLE patients and correlate with disease activity index and lupus nephritis, indicating that these cells can be used as potential biomarkers for the disease (Sachinidis et al., 2025). In SSc, ABC-like cells were found to be expanded and contribute to vascular complications of the disease (Kourkouni et al., 2024). In addition, in the case of multiple sclerosis (MS), ABCs expand and exhibit a significantly upregulated inflammatory cytokine profile, in terms of mRNA expression (SoRelle et al., 2025). Beyond autoimmunity, chronic infections such as HIV, hepatitis C and malaria, induce similar ABC expansions, linking persistent immune activation to their emergence (Portugal et al., 2017; Knox et al., 2017).

Regarding ABCs, it is important to mention that specific targeting of this population of B cells can alleviate symptoms in various autoimmune diseases, including SLE, RA and MS (Sachinidis et al., 2024). Currently therapeutic interventions targeting BAFF (e.g., belimumab) and/or CD20 (e.g., rituximab) reduce ABC frequencies in the human blood of SLE patients, indicating their implementation in autoimmunity pathogenesis (Sachinidis et al., 2020; Ramsköld et al., 2019). Additional important ABC reducing therapies include an extended portfolio of IRF5 inhibitors (genetic and/or chemical inhibition), JAK inhibitors (such as baricitinib), triggering of adenosine receptor 2a (A2a) via A2a agonists, TNF inhibitors, administration of tocilizumab (antagonist of IL-6 receptor) and ROCK-kinase inhibitors (such as fasudil) (Sachinidis et al., 2025; Sachinidis et al., 2024).

## Double negative B cells (DN)

Double negative (DN) B cells are a heterogeneous population, defined by the absence of IgD and CD27 markers (Sachinidis and Garyfallos, 2021). These cells are expanded in the elderly and, moreover, seem to showcase different immune functions in different pathological conditions (refers to infectious diseases and autoimmunity) (Sachinidis and Garyfallos, 2021). For instance, in case of HIV infection and/or malaria, a proportion of DN B cells displays an exhausted phenotype, while in SLE cases these cells are considered as the main source of autoantibody secretion (Sachinidis and Garyfallos, 2021; Jenks et al., 2018; Beckers et al., 2023).

DN B cells are comprised of at least four subsets and are categorized into subgroups, based on the expression of CXCR5 chemokine, CD11c integrin and transcription factor T-bet: DN1 (CXCR5^+^CD11c^−^T-bet^−^), DN2(CXCR5^−^CD11c^+^T-bet^+^), DN3 (CXCR5^−^CD11c^−^T-bet^low^), and DN4 (CXCR5^+^CD11c^−^T-bet^−^) (Sachinidis and Garyfallos, 2021; Somers et al., 2022; Castleman et al., 2022). DN1 cells are enriched in healthy elderly individuals and are primarily associated with immunosenescence (Colonna-Romano et al., 2009). Of note, DN1 appear relatively non-pathogenic and lack a strong T-bet–driven phenotype (Sachinidis and Garyfallos, 2021; Colonna-Romano et al., 2009; Somers et al., 2022; Castleman et al., 2022). DN2 cells, on the other hand, are highly responsive to TLR7 and have been strongly associated with extrafollicular plasmablast differentiation, particularly in inflammatory settings, and are strongly linked to active SLE, especially in African American women with lupus nephritis (Jenks et al., 2018). Interestingly, DN2 is considered to be the only DN subset highly expressing T-bet (Sachinidis and Garyfallos, 2021; Jenks et al., 2018; Somers et al., 2022; Castleman et al., 2022). Initially, it was presumed that these cells were lupus specific (Jenks et al., 2018). Subsequently, however, their presence was also confirmed in other rheumatic conditions, including rheumatoid arthritis (Wing et al., 2023). As far as DN3 cells are concerned, this subset constitutes a newly recognized DN subset that has been linked to extrafollicular immune activation and has been associated with severe COVID-19 and hypoxic conditions (Sachinidis and Garyfallos, 2021; Castleman et al., 2022). In addition, the population has also recently been implicated in autoimmune fibrosis in the context of IgG4-related disease, where it infiltrates inflamed tissues (Allard-Chamard et al., 2023), as well as in SLE, where it shows a significant correlation with disease activity (Chizzolini et al., 2024). Lastly, DN4 cells remain a poorly defined population. According to some studies, though, the aforementioned DN cells are closely related to allergic reactions (Sachinidis and Garyfallos, 2021; Somers et al., 2022; Castleman et al., 2022). Overall, although DN subsets - particularly DN2 and DN3 - are strongly associated with extrafollicular immune responses, their developmental trajectories are likely context-dependent and may vary across disease settings (Sachinidis and Garyfallos, 2021; Jenks et al., 2018; Somers et al., 2022; Castleman et al., 2022).

Transcriptomic analyses confirm that DN2 cells share common features with ABCs, including T-bet and CD11c expression (Jenks et al., 2018), although these similarities may vary depending on the inflammatory or pathological context. In contrast, DN3 cells display a distinctive signature, characterized by a strong signature of proliferation and unfolded protein response, along with lowest - among DN subsets - levels of CD22, CD72, CD69 and BAFFR expressions (Castleman et al., 2022; Allard-Chamard et al., 2023). DN1 are found in healthy elderly individuals, while DN4 seem to be strongly associated with allergies (Colonna-Romano et al., 2009; Somers et al., 2022; Castleman et al., 2022). The former have been shown to transcriptomically resemble memory B cells, while the latter express genes linked to the Notch signaling pathway and protein ubiquitination, and thus are distinguished from DN1 (Allard-Chamard et al., 2023). However, both two sub-types express CXCR5^+^ chemokine, in contrast to DN2 and DN3 cells (Sachinidis and Garyfallos, 2021). Interestingly, DN2 and DN3 cells are those that are mostly associated with autoimmune diseases (Jenks et al., 2018; Allard-Chamard et al., 2023; Chizzolini et al., 2024). In total, these differences in function and transcriptomic characterization suggest that every DN sub-population is a complete discrete entity (Chung et al., 2023).

Regarding disease involvement, DN B cells, similar to ABCs, expand in systemic autoimmune diseases and infections (Sachinidis and Garyfallos, 2021; Jenks et al., 2018; Portugal et al., 2017; SoRelle et al., 2025; Beckers et al., 2023). For instance, in MS, both ABCs and DN have been reported to expand and exhibit some pro-inflammatory characteristics (Claes et al., 2016). In SLE, furthermore, both populations increase in numbers and seem to drive disease pathogenesis via differentiating into plasma cells and producing autoantibodies (Jenks et al., 2018; Wang et al., 2018). In infectious diseases, such as COVID-19 and malaria, DN expansion has also been reported and linked to poor clinical outcomes (Woodruff et al., 2020; Sutton et al., 2021). Importantly, these observations suggest that the functional relationship between ABCs and DN B cells may depend on the specific inflammatory or pathological context.

## Relationship of ABCs to DN B cells

ABCs and DN B cells share many similarities, and are therefore often considered related populations (Sachinidis et al., 2020). Both subsets have been reported to expand in elderly individuals (Hao et al., 2011; Colonna-Romano et al., 2009), during infections (Portugal et al., 2017; Beckers et al., 2023), and in autoimmune diseases (Jenks et al., 2018; Wang et al., 2018). Regarding immunophenotype, they exhibit overlapping features, including expression of CD11c and/or T-bet (Sachinidis and Garyfallos, 2021; Jenks et al., 2018; Wang et al., 2018). Moreover, both populations display similar activation requirements, which involve IFN-γ, IL-21, and TLR7 or TLR9 signaling (Jenks et al., 2018; Wang et al., 2018; Rubtsov et al., 2011; Naradikian et al., 2016). Supporting this parallel, pharmacological agents that reduce ABC frequencies in human blood also appear to decrease DN B cell percentages (Sachinidis et al., 2024). Notably, in both healthy individuals and patients with lupus, ABC frequencies correlate with DN B cell frequencies (Sachinidis et al., 2025; Chizzolini et al., 2024). Collectively, these findings indicate that ABCs and DN B cells are highly similar at first glance. However, the extent of these similarities may vary depending on the inflammatory or pathological context, highlighting that their functional and transcriptomic relationship is not absolute.

Beyond antibody secretion, ABCs have been shown to produce pro-inflammatory cytokines and/or release chemokines, serve as antigen-presenting cells with strong phagocytic capacity, and also contribute to T-cell activation (Xie et al., 2025). Similarly, DN B cells - particularly the DN2 subset - exhibit comparable effector functions: upon stimulation, they produce pro-inflammatory cytokines, express antigen-presentation markers such as HLA-DR and CD86, and can activate T cells (Li et al., 2021; Moysidou et al., 2023). These findings indicate that both ABCs and DN B cells are not merely antibody precursors, but can perform broader immune functions, with their activity often depending on the inflammatory or pathological context.

Despite their similarities, ABCs differ from DN B cells in several key aspects. Some ABCs highly express the memory marker CD27 (Rubtsov et al., 2011), which is absent from DN B cells (Jenks et al., 2018; Colonna-Romano et al., 2009). Although the majority of ABCs are class-switched B cells, predominantly expressing IgG or IgA, single-cell RNA-seq analyses reveal that this population also contains unswitched IgD + cells (Ambegaonkar et al., 2022). Furthermore, DN1, DN3 and DN4 subsets (which are less well-characterized than DN2) lack expression of CD11c and T-bet (Somers et al., 2022; Castleman et al., 2022; Chung et al., 2023), two defining features of ABCs (Wang et al., 2018). With respect to autoantibody production, a hallmark of ABC function (Cancro, 2020; Rubtsov et al., 2011), only DN2 cells among DN B cell subsets efficiently differentiate into plasma cells (Jenks et al., 2018). Regarding their origins, ABCs are commonly thought to include GC–experienced cells, although alternative developmental pathways have been proposed (Cancro, 2020; Hao et al., 2011; Li et al., 2023), whereas DN B cells have been strongly associated with extrafollicular differentiation, particularly under inflammatory or autoimmune settings (Jenks et al., 2018; Chizzolini et al., 2024; Woodruff et al., 2020), suggesting that their relationship with ABCs may be context-dependent. Lastly, according to a comparative transcriptomic analysis, ABCs are distinct from other CD11c^+^ B cell populations, such as DN2, as they display an elevated expression in multiple cytokines and chemokines, which are not detected as increased in the other CD11c^+^ subsets (Maul et al., 2021).

In recent literature, the term “ABCs” refers to murine B cells, whereas in humans the “DN B cells” - particularly DN2 cells - are regarded as their corresponding counterparts (Chung et al., 2023; Ricker et al., 2021; Satterthwaite, 2021). This scenario is plausible, although ABC cells (or at least ABC-like cells) have also been reported in humans (Wang et al., 2018; Portugal et al., 2017; Rubtsov et al., 2011; Kourkouni et al., 2024; Sachinidis et al., 2025; Claes et al., 2016). ABCs represent a heterogeneous B-cell population (Nickerson et al., 2023), and several related circulating and/or splenic subsets have been described in both mice and humans (Phalke and Marrack, 2018). Similarly, DN B cells are heterogeneous, with four discrete subsets identified to date (Somers et al., 2022; Castleman et al., 2022). Considering the resemblances and differences between ABCs and DN B cells (Table 1), we propose that one ABC subset - lacking IgD and CD27 expression - closely corresponds to DN2 cells, though this relationship may be influenced by the specific inflammatory or pathological environment. Memory (CD27^+^) and phenotypically naïve-like (IgD+) ABCs cannot be classified as DN cells (Jenks et al., 2018; Colonna-Romano et al., 2009; Rubtsov et al., 2011; Ambegaonkar et al., 2022), and conversely, DN B cells lacking CD11c and T-bet cannot be classified as ABCs (Wang et al., 2018; Somers et al., 2022; Castleman et al., 2022; Chung et al., 2023). Notably, IgD+ ABCs are not considered naïve cells, as their population carries somatic hypermutations and is clonally related to IgD-cells, indicating prior antigen experience (Maul et al., 2021). As consensus has not yet been reached regarding the optimal immunophenotypic markers defining ABCs, we suggest that this specific B-cell population should be regarded not only as murine, but also as human, being closely related to DN2 cells (Jenks et al., 2018).

**TABLE 1:** Phenotypic and functional characteristics of age-associated B cells and double-negative B cells.

​	Atypical B cells		​
Feature	Age-associated B cells (ABCs)	Double-negative B cells (DN B cells, CD27^−^IgD^−^)	Notes on similarities/Differences
Surface markers	CD19^+^, CD11c^+^, T-bet^+^, often CD21^−^	CD19^+^, CD27^−^, IgD^−^, sometimes CD11c^+^ and T-bet^+^ (especially DN2 subset)	DN2 subset shares marker profile with ABCs; other DN subsets do not
Transcription factors	T-bet (key driver of ABC differentiation)	DN2 subset expresses T-bet; DN1, DN3 and DN4 subsets do not	ABCs defined by T-bet; DN B cells are heterogeneous
Origin/Development	Arise during aging, chronic viral infections, or autoimmune responses; often derived from naive or memory B cells under inflammatory conditions	Can arise from naive B cells or atypical activation; expanded in aging, SLE, chronic infections	Both expand with age and chronic immune stimulation
Functional properties	Strong antigen-presenting capacity, pro-inflammatory cytokine production, can differentiate into autoantibody-producing plasma cells	DN2 subset: hyper-responsive to TLR7 stimulation, autoantibody production; DN1: less active, DN3 and DN4: less well characterized	Functional overlap mainly with DN2 subset; both contribute to autoimmunity
Tissue distribution	Spleen, peripheral blood, lymph nodes	Peripheral blood, sometimes inflamed tissues	ABCs more prominent in secondary lymphoid organs
Role in disease	Autoimmune diseases (SLE, RA, etc.), chronic infections, aging	Autoimmunity (SLE, RA, etc.), chronic infections, aging	Overlapping pathological roles
Cytokine responsiveness	Responsive to IFN-γ, TLR7/9, IL-21	DN2: responsive to TLR7, IFN-γ, IL-21; DN1 less responsive, DN3 and DN4: less well characterized	DN2 and ABCs share cytokine responsiveness
Proliferation/Activation	Low at baseline, activated under inflammatory signals	DN2: hyperactive; DN1: more quiescent DN3 and DN4: less well characterized	DN2 subset functionally closest to ABCs

Interestingly, while Knox et al. (2025) represent an important refinement of murine ABC definitions (Knox et al., 2025), further observations across studies highlight ongoing heterogeneity of ABCs. In more detail, Knox et al. report relative resistance of ABCs to anti-BLyS and anti-CD20 depletion in murine lupus models (Knox et al., 2025), whereas Ramsköld et al. (2018) and Faustini et al. (2022) found sensitivity of ABCs to these same interventions, in SLE patients (Ramsköld et al., 2019; Faustini et al., 2022). Clearly, such observations further highlight the continuing need for ABC characterization in both human and murine systems.

Similar to the case of ABCs, the optimal characterization of DN B cells is of utmost importance. This need is reflected, for example, in the observation that a DN B cell population with cytoplasmic FOXO1 has been identified in patients with SLE (Hritzo Ahye and Golding, 2018). However, it is still unknown whether this population corresponds to any known DN subset observed in SLE patients - such as DN2 or DN3 - or whether it represents another distinct population (Sachinidis and Garyfallos, 2021; Jenks et al., 2018; Chizzolini et al., 2024; Hritzo Ahye and Golding, 2018). Of note, FOXO1 is a transcription factor that plays a key role in B cell development (Sander et al., 2015).

## Conclusion

ABCs constitute a heterogeneous population of B cells, comprising CD27^+^ B cells, IgD+ B cells and–predominantly–IgD-CD27^−^ (DN) B cells (Tangye, 2023). Compelling evidence from immunophenotypic, functional, and transcriptomic analyses indicates that ABCs with an IgD^−^CD27^−^ phenotype are closely related to the DN2 B cell subset, which has been well-characterized in lupus (Jenks et al., 2018; Sachinidis et al., 2023). Notably, the extent of this relationship may vary depending on the inflammatory or pathological context.
